# Prognostic Model of Colorectal Cancer Constructed by Eight Immune-Related Genes

**DOI:** 10.3389/fmolb.2020.604252

**Published:** 2020-11-27

**Authors:** Shuting Wen, Long He, Zhuotai Zhong, Hong Mi, Fengbin Liu

**Affiliations:** ^1^The First Clinical College, Guangzhou University of Chinese Medicine, Guangzhou, China; ^2^Department of Gastroenterology, The First Affiliated Hospital of Guangzhou University of Chinese Medicine, Guangzhou, China

**Keywords:** colorectal cancer, immune-related gene, prognostic model, bioinformatics, immune microenvironment

## Abstract

**Background:**

Colorectal cancer (CRC) is a common malignant tumor of the digestive tract with a high mortality rate. Growing evidence demonstrates that immune-related genes play a prominent role in the occurrence and development of CRC. The aim of this study was to investigate the prognostic value of immune-related genes in CRC.

**Methods:**

Gene expression profiles and clinical data of 568 CRC and 44 non-tumorous tissues were obtained from The Cancer Genome Atlas (TCGA) database. First, we performed a differentially expressed gene (DEG) analysis and univariate Cox regression analysis to determine the DEGs associated with overall survival. Gene ontology (GO) and Kyoto Encyclopedia of Genes and Genomes (KEGG) pathway enrichment analyses were subsequently performed for prognostic immune-related genes. Then, a multivariate Cox regression analysis was performed to establish the immune prognostic model and identify the independent prognostic factors of CRC. Next, *in vitro* experiments were done to further validate the model. Finally, we analyzed the correlation among immune-related genes, clinical traits, and immune cell infiltration.

**Results:**

In total, 3,702 DEGs were obtained, and 338 prognostic immune-related genes were identified. Among them, 45 genes were significantly correlated with the prognosis of CRC patients. A TF-mediated network was set up to explore its internal mechanism. GO and KEGG analyses further illustrated that these genes were enriched in immune-and inflammatory-related pathways. Then, a prognostic prediction model composed of eight immune-related genes (SLC10A2, UTS2, FGF2, UCN, IL1RL2, ESM1, ADIPOQ, and VIP) was constructed. The AUC of the ROC curve for 1, 3, 5, and 10 years overall survival (OS) was 0.751, 0.707, 0.680, and 0.729, respectively. The survival analysis suggested that the OS of the high-risk group was significantly poorer than that of the low-risk group. Meanwhile, *in vitro* assays revealed that ESM1 and SLC10A2 exert opposing roles in colon cancer cell proliferation, validating the accuracy of the model. The correlation analysis indicated that immune cell infiltration was positively related to the model.

**Conclusion:**

This study screened prognosis-related immune genes and developed a prognostic prediction model of CRC. These findings may help provide potential novel prognostic biomarkers and therapeutic targets for CRC. At the same time, the understanding of the CRC immune microenvironment status was deepened.

## Introduction

Colorectal cancer (CRC) is known as the third most common cancer globally and has the second-highest leading number of cancer-related deaths worldwide (Keum and Giovannucci, 2019). Every year, an estimated 9,00,000 people die from CRC all over the world, and more than 1.8 million people are diagnosed with CRC (Dekker et al., 2019; Safiri et al., 2019). The morbidity and mortality rate of CRC has been rising in Asia (Onyoh et al., 2019), which has become a major public health problem. Because the early symptoms of CRC are not obvious, most patients are overlooked (Or et al., 2017). They are often diagnosed in intermediate or late stages and have poor prognoses with the symptoms of hematochezia, abdominal pain, and altered bowel habits, as well as emaciation (Dekker et al., 2019). Despite recent progress in testing and treatments, the overall prognosis for patients with CRC remains poor because biomarkers for early detection and prognosis prediction are lacking (Keum and Giovannucci, 2019). Thus, it is essential to explore valuable diagnostic and prognostic factors, as well as potential effective therapeutic targets.

The immune system is important for the progression of cancer (Patel and Minn, 2018). Immunotherapy is a new kind of therapy for cancer and other chronic conditions that targets the human immune system (Thyer et al., 2013; Zhang et al., 2019). It is commonly accepted that CRC is a clinically heterogeneous and immunogenic disease (Leman et al., 2018). There are various therapeutic approaches for CRC, such as surgery, radiotherapy, and chemotherapy (Dekker et al., 2019). Furthermore, targeted therapy and immunotherapy are more conducive to the treatment of CRC patients (Ganesh et al., 2019). Immune cells play a key role; from tumor growth to their invasion and metastasis (Ciardiello et al., 2019). An increasing number of studies have found that a large number of immune-related genes are associated with the development of CRC (Cereda et al., 2016; Yu et al., 2019). In recent studies, prognostic-related genes associated with CRC patients were extracted according to their gene expression profile (Sun et al., 2018; Zuo et al., 2019), while Bai et al. (2020) obtained immune-related genes with the prognosis in CRC patients. Notwithstanding, there are currently no immune-related differentially expressed genes (DEGs) to systematically evaluate the prognosis of CRC.

In the current study, we applied the gene expression data and the corresponding clinical information of CRC patients from a large-scale sequencing database. By using a bioinformatics analysis, prognostic immune-related DEGs were identified. Gene ontology (GO) and enrichment analyses were conducted to reveal the mechanism of the immune response in CRC. Subsequently, we constructed a prognostic model of patients with CRC by integrating the key immune-related genes. The purpose of this study was to provide new biomarkers for the prediction of the CRC prognosis and tumor immune microenvironment. These observations may provide new insights toward novel therapeutic targets for CRC.

## Materials and Methods

### Data Collection

**FIGURE 1:**
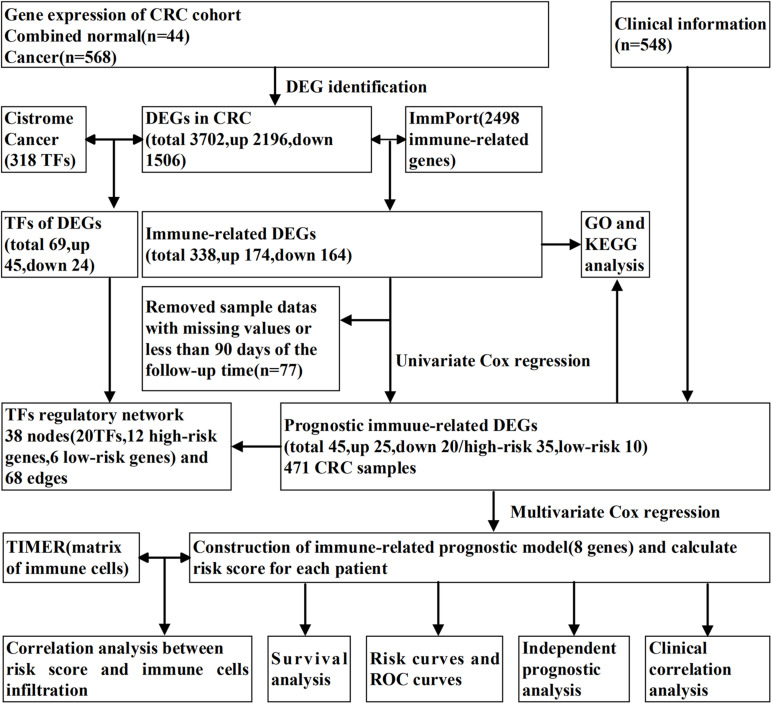
Experimental flowchart for immune-related prognostic model of CRC.

The study scheme is illustrated in Figure 1. Gene expression matrix data of CRC samples were obtained from The Cancer Genome Atlas (TCGA) data portal^1^(up to February 26, 2020). Transcriptome information for cancers with “colon” or “rectum” as the primary site from a “TCGA-COAD” and “TCGA-READ” project were included. HT-Seq FPKM data of 568 CRC and 44 non-tumorous tissues were downloaded for further analysis. From the database, we also searched the clinical information of the related patients, including survival information, age, gender, tumor stage, and histology classification. A total of 2,498 immunologically relevant genes were retrieved from the Immunology Database and Analysis Portal (ImmPort)^2^ database (Bhattacharya et al., 2018). For exploring the regulatory mechanism of immune genes related to CRC, we downloaded 318 transcription factors (TFs) via Cistrome Cancer^3^ (Mei et al., 2017), which is a comprehensive resource for genetic research in cancers.

### Differentially Expressed Gene (DEG) Identification

From TCGA we obtained the expression matrix data of 13,673 RNAs in CRC. The DEGs between CRC and adjacent normal tissues were identified using the Wilcoxon test method with the “limma” (Ritchie et al., 2015) package in R software (version 3.6.2). Meanwhile, immune-related DEGs and TFs in CRC were screened, with the conditions of | LogFC| > 1 and a false discovery rate (FDR) of < 0.05 was set as significant. The upregulated and downregulated genes were visualized on volcano plots, while heatmaps were generated to show the expression profiles of significant immune-related DEGs and TFs using the “pheatmap” package. To identify the biological properties of immune-related DEGs, GO, and KEGG pathway enrichment analyses were conducted using the “clusterProfiler,” “enrichplot,” and “ggplot2” packages. Enrichment results with an FDR (false discovery rate) of < 0.05 were recognized as significant functional categories.

### Identification of Prognostic Immune-Related Genes and Construction of the Network With TFs

To make the results more accurate, patients with a follow-up time of more than 90 days were included in the survival analyses. Univariate Cox regression was applied to examine the prognostic value of immune-related DEGs in CRC patients using the “survival” package. *P*-values of < 0.05 were considered as significant and shown by a forest plot. Genes with a hazard ratio (HR) > 1 were identified as high-risk genes, while a HR < 1 was identified as low risk. The potential biological functions of these prognostic immune-related DEGs were further performed by GO and KEGG enrichment analyses. To determine the mechanisms between the survival-related immune genes and transcription factors, a Pearson’s correlation test was conducted in the R software. Both the correlation coefficient (cor) and *P*-value were estimated. The study set the filtering criteria as | cor| > 0.4 and *P* < 0.001. It was considered that cor > 0 showed a relationship of positive correlation, while cor < 0 indicated a negative correlation. The regulatory network between TFs and prognostic immune-related genes was constructed and visualized via Cytoscape 3.7.1.

### Construction of the Prognostic Immune-Related Gene Signature for CRC

After acquiring the prognostic immune-related genes of CRC with a *P*-value of < 0.01, a multivariate Cox regression analysis was implemented to develop a prognostic model using the “survival” package. The model was utilized to evaluate the connection of overall survival (OS) and immune-related genes. According to the model, risk scores were calculated, and patients were divided into high and low risk groups on the basis of the median risk score. For validation of the model, a Kaplan-Meier log-rank analysis was implemented via the “survival” and “survminer” packages. Then, the receiver operating characteristic (ROC) curve was generated using the “survival ROC” package, as well as a risk plot performed using the “pheatmap” package.

### Independent Prognostic Analysis and Clinical Correlation Analysis

Univariate and multivariate independent prognostic analyses were utilized by the “survival” package. The aim was to evaluate whether the risk score is an independent prognostic factor that is associated with overall survival. A clinical relevance analysis was conducted using the “beeswarm” package to assess the correlation between prognostic immune-related DEGs and clinical traits.

### Correlation Analysis Between Risk Score and Immune Cell Infiltration

To examine the connection between the immune-related signature and immune cell infiltration, we obtained the matrix of immune cells from the Tumor Immune Estimation Resource (TIMER)^4^, which is a useful web server for the systematic analysis of immune infiltrates in cancer. Users can predict the abundancies and proportion of six immune cell subsets (B cells, CD4^+^ T cells, CD8^+^ T cells, dendritic cells, macrophages, and neutrophils). A Pearson’s correlation test was utilized in R to explore the correlation between the risk scores and tumor infiltrating immune cells.

### Cell Lines and Cell Transfection

Human CRC cell lines HCT-116 were cultured in RPMI-1640 medium, while SW-480 cell lines were cultured in DMEM medium. Both media contained 10% fetal bovine serum (FBS; Gibco, United States), 100 U/mL penicillin, 100 μg/mL streptomycin. ESM1-specific siRNA, SLC10A2-specific siRNA, and control siRNA were purchased from the Generay Biotech (Shanghai) Co., Ltd. The siRNA sequence targeting ESM1 were as follows: sense siRNA:5′-CUC UCA CGG AGC AUG ACA UTT-3′; and antisense siRNA: 5′-AUG UCA UGA UCC GUG AGA GTT-3′. The targeted sequences of SLC10A2 were sense siRNA: 5′-CCA AAG CGC CUG GAU CAU UTT-3′; and antisense siRNA:5′-AAU GAU CCA GGC GCU UUG GTT-3′. ESM1 and SLC10A2 siRNA transfection was performed with Lipofectamine 2000 (Invitrogen) in 6-well plates following the manufacturer’s protocol.

### Cell Proliferation Assay

The cell proliferation and viability assays were assessed by Cell Counting Kit-8 (CCK-8, Biosharp, China). Cells were collected at 24 h after transfection and were inoculated into 96-well culture plates (2 × 10^3^ cells/well). Subsequently, 10 μL of CCK-8 was added at different time points to each well and incubated at 37°C for 2 h. The optical density (OD) was measured at 450 nm with a microplate reader (Thermo Fisher Scientific, Waltham, MA, United States) and the cell viability was calculated.

### Statistical Analysis

All analyses were conducted using R version 3.6.2 and GraphPad Prism 8.0 (GraphPad Software, San Diego, United States). Significance was determined as ^∗^*p* < 0.05, ^∗∗^*p* < 0.01 unless otherwise specified.

## Results

### Differentially Expressed Immune-Related Genes in CRC

**FIGURE 2:**
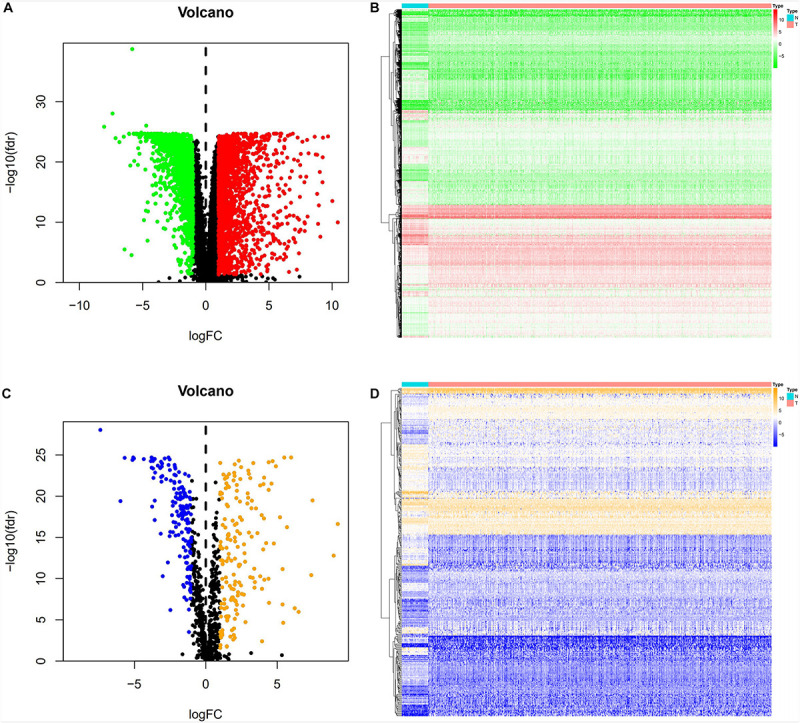
DEGs and immune-related DEGs in CRC. **(A)** Volcano plot of DEGs. The red dots represent the upregulated genes and the green dots represent the downregulated genes in CRC. Black dots represent no statistical significance. **(B)** Heatmap of DEGs. The process from low to high expression of each gene is shown by the color from green to red. **(C)** Volcano plot of immune-related DEGs. The orange dots indicate the upregulated genes and the blue dots indicate the downregulated immune-related genes in CRC. **(D)** Heatmap of immune-related DEGs. Orange represents higher expression while blue represents lower expression in CRC.

A total of 3,702 genes (2,196 upregulated and 1,506 downregulated) were considered as DEGs in CRC tissues compared with adjacent normal tissues. Among them, there were 338 immune−related genes (174 upregulated and 164 downregulated) that were identified as differentially expressed. The distribution of DEGs and immune−related DEGs between CRC and normal tissues were visualized by volcano plots (Figures 2A,C). The heatmaps revealed the expression changes of genes and immune-related genes, which can be obviously distinguished between CRC and a normal sample (Figures 2B,D).

**FIGURE 3:**
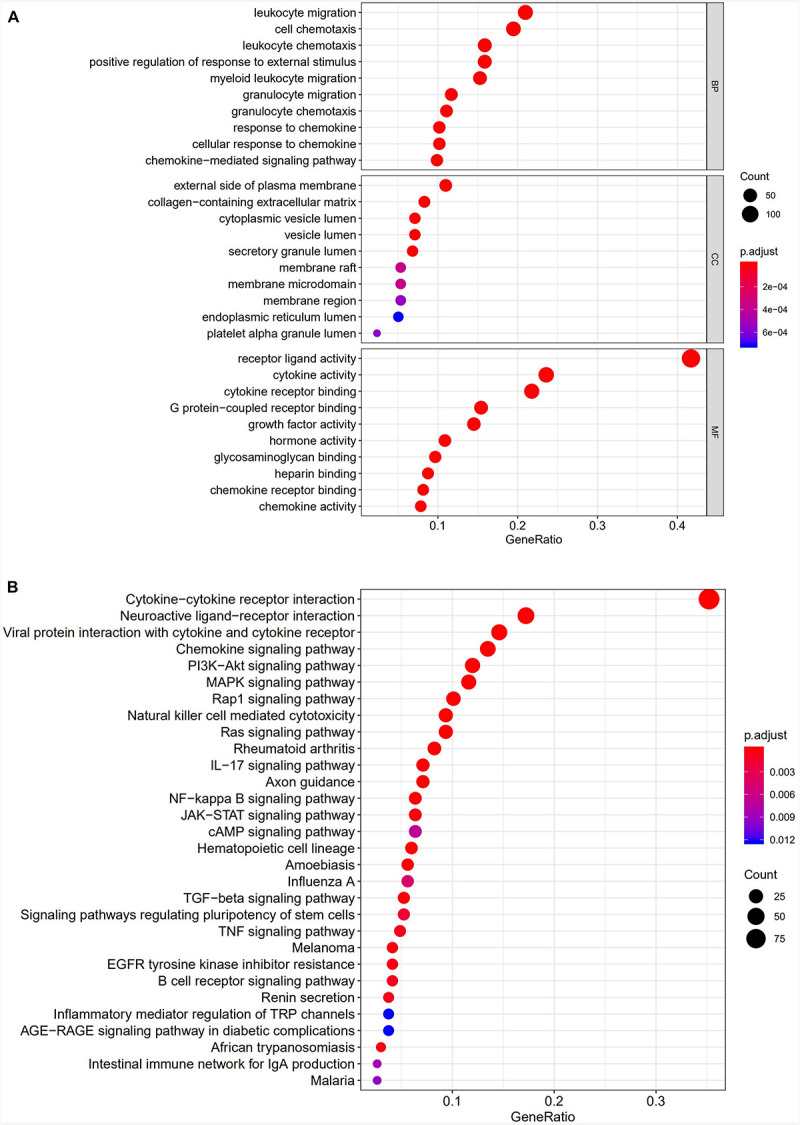
Gene functional enrichment analysis of immune-related DEGs. **(A)** The top 10 significant biological process, cellular component, and molecular function terms of the GO analysis. **(B)** The top 30 most significant KEGG pathways.

All 338 immune-related DEGs were analyzed by GO and KEGG analyses. The results indicated that the most enriched biological processes (BP) were leukocyte migration, cell chemotaxis, and leukocyte chemotaxis. For cellular components (CC), immune-related DEGs were mainly involved in the external side of the plasma membrane, collagen-containing extracellular matrix, and cytoplasmic vesicle lumen. The major functional categories in molecular function (MF) were receptor ligand activity, cytokine activity, and cytokine receptor binding (Figure 3A). The KEGG pathway enrichment analysis revealed that immune-related genes were particularly enriched in cytokine–cytokine receptor interaction and neuroactive ligand–receptor interaction, as well as in viral protein interaction with cytokines and cytokine receptors (Figure 3B).

### Screening of Prognostic Immune-Related DEGs in CRC

**FIGURE 4:**
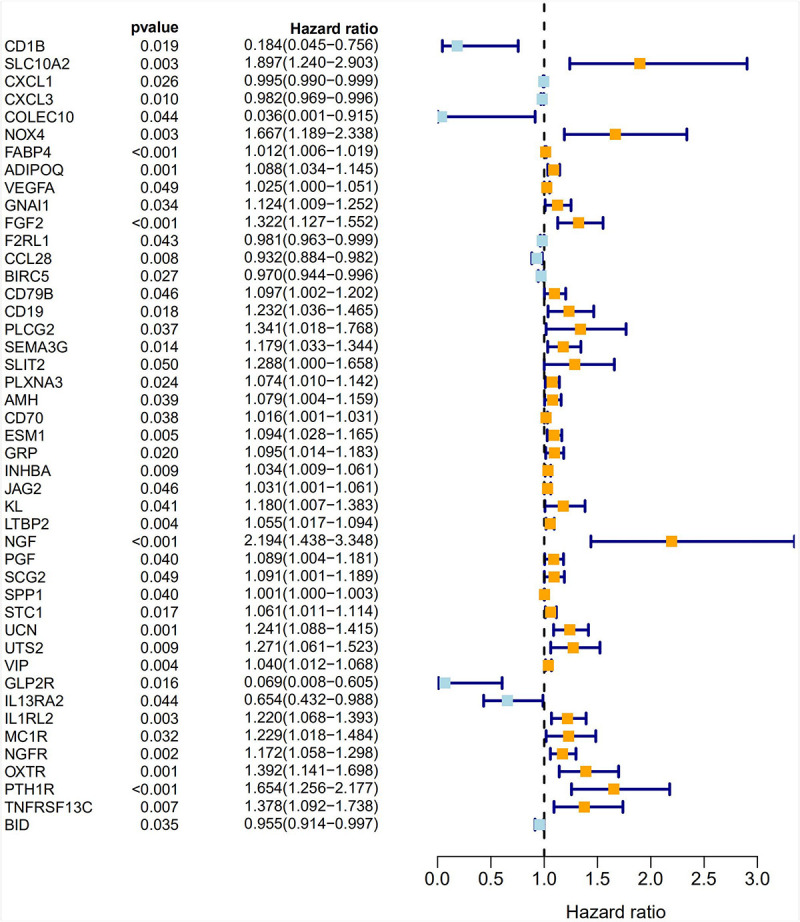
Forest plot of 45 prognostic immune-related DEGs in CRC. Hazard ratios (HR) and corresponding 95% confidence intervals (CI) were estimated using the univariate Cox regression model. The orange modules represent high-risk genes and the light blue modules represent low-risk genes in CRC.

**FIGURE 5:**
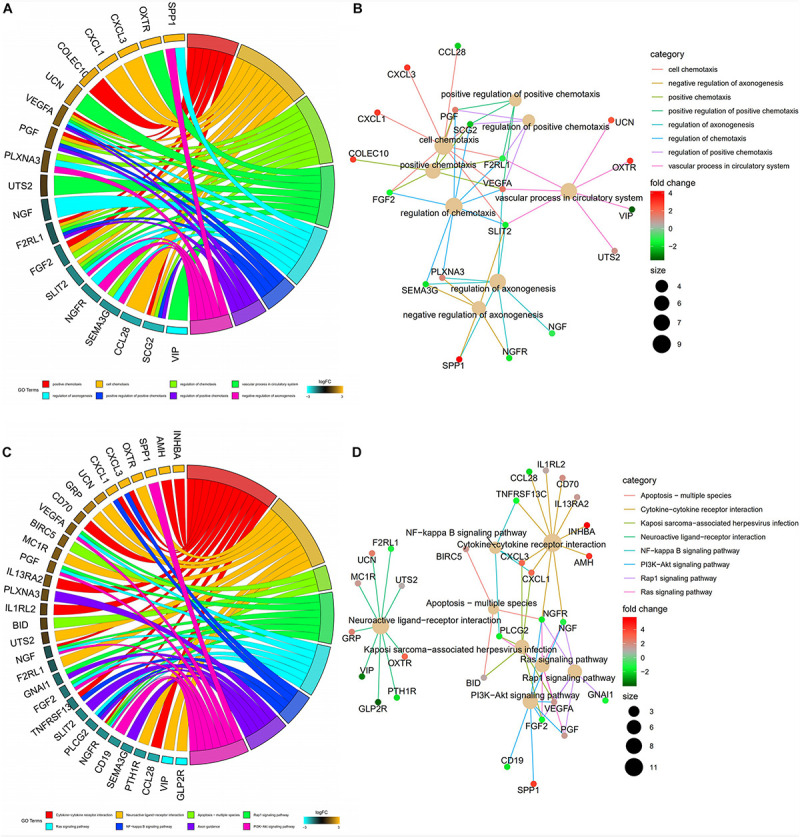
Functional enrichment analysis of prognostic immune-related DEGs. **(A)** The top 8 significant biological process terms of the GO analysis. Genes and their logFC expression in each enriched gene ontology term are shown. **(B)** Construction of the BP terms-genes network. Degrees were visualized by the size of the nodes, while the interaction between BP terms and genes were represented by the edge. **(C)** Top 8 enriched KEGG pathways for the prognostic immune-related genes. **(D)** Construction of the enrichment pathways-genes network.

A univariate Cox analysis was performed on 338 immune-related differential genes in 471 CRC samples, and 45 genes related to overall survival were obtained (Figure 4 and Supplementary Table 1). Among them, 35 genes were identified as high-risk genes, while 10 were low-risk genes. The GO analysis revealed that these prognostic genes were mainly involved in positive chemotaxis, cell chemotaxis, and the regulation of chemotaxis (Figures 5A,B). The most enriched KEGG pathways were cytokine–cytokine receptor interaction, neuroactive ligand–receptor interaction, and apoptosis-multiple species (Figures 5C,D). All these pathways were significantly related to the development of CRC.

### TF Regulatory Network

**FIGURE 6:**
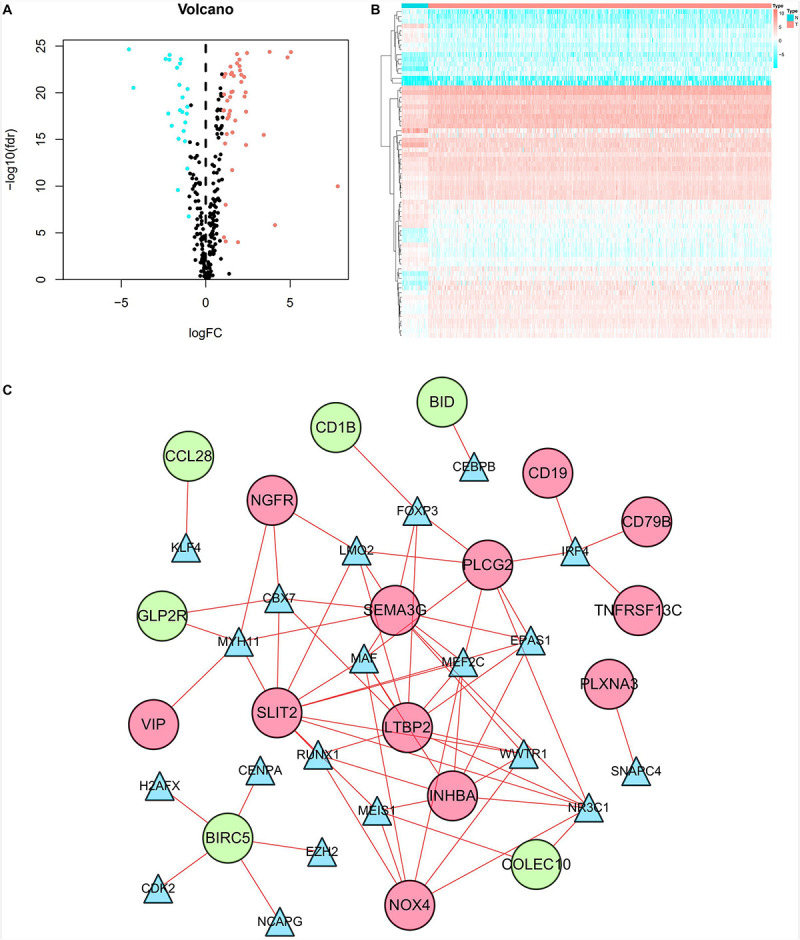
TF regulatory network. **(A)** Volcano plot of differentially expressed TFs. The salmon dots represent the upregulated TFs and the cyan dots represent the downregulated TFs in CRC. Black dots represent no statistical significance. **(B)** Heatmap of differentially expressed TFs. The progression from low to high expression of TFs is shown by the color from cyan to salmon. **(C)** Construction of the network with TFs and prognostic immune-related differentially expressed genes. The blue triangles, red circles, and green circles represent TFs, high-risk genes, and low-risk genes, respectively.

Our results showed that 69 TFs (45 upregulated and 24 downregulated) were differentially expressed between CRC and normal tissues. The distribution of TFs was represented by a volcano plot (Figure 6A), while the expression changes of the TFs were revealed by a heatmap (Figure 6B). We also identified 9 prognosis-related TFs using the univariate Cox regression analysis, including TCF7L1, SALL4, ELF5, SNAPC4, WWTR1, FOSL1, TEAD4, FOXM1, and NCAPG (Supplementary Table 2). Relevant TFs and immune-related genes were selected by correlation analysis, and a regulatory network was established to elucidate the mechanism of immune-related genes in CRC. The network consists of 38 nodes (20 TFs, 12 high-risk, and 6 low-risk prognosis-related genes) with 68 edges (Figure 6C). It was shown that NR3C1 regulated most of the high-risk genes. Meanwhile, all the TFs in the network had positive relationships with the immune-related genes.

### Construction of Immune-Related Prognostic Model for CRC

**FIGURE 7:**
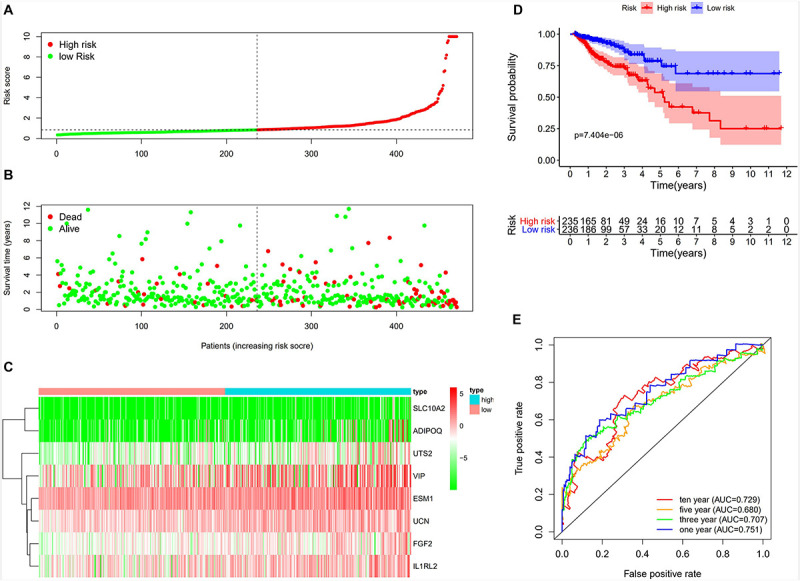
Construction of the immune-related prognostic model for CRC. **(A)** The risk score distribution of CRC patients in the TCGA database. The progression from low-to high-risk scores is shown by the color from green to red. **(B)** Survival status and survival time of each CRC patient. **(C)** Heatmap of the expression of eight prognostic immune–related genes in CRC patients. **(D)** Survival analysis and overall survival rate of the low-risk and high-risk groups. **(E)** Time-dependent ROC curves of OS for the prognostic model.

By utilizing the univariate Cox analysis, 13 prognostic immune-related genes were filtered. A total of eight genes were finally identified to develop a prognostic model after performing six multivariate Cox analyses (Table 1). Based on the expression levels of genes and the corresponding Cox regression coefficient, the formula was shown as: the risk score = (expr_*SLC1*0A2_^∗^ 0.633) + (expr_*ADIPOQ*_^∗^ 0.087) + (expr_FGF2_^∗^ 0.326) + (expr_*ESM*1_^∗^ 0.106) + (expr_*UCN*_^∗^ 0.283) + (expr_*UTS*2_^∗^ 0.333) + (expr_*VIP*_^∗^ 0.044) + (expr_*IL*1R*L*2_^∗^ 0.221). All eight genes were high-risk genes, with HRs of > 1. The CRC patients were divided into a high-risk group (*n* = 235) and a low-risk group (*n* = 236) on the basis of the median score of 0.837 (Figure 7A). A survival status overview and eight-gene expression heatmap were established (Figures 7B,C). The Kaplan-Meier analysis confirmed that patients in the high-risk group had poorer overall survival (OS) compared to those in the low-risk group. The 5 years survival rate of the high-risk group was 0.499 (95% CI: 0.376–0.664), while that of the low-risk group was 0.744 (95% CI: 0.630–0.880) (Figure 7D). The AUC of the ROC curve for 1, 3, 5, and 10 years OS was 0.751, 0.707, 0.680, and 0.729, respectively. The results indicate that the prognostic model has good sensitivity and specificity (Figure 7E).

**TABLE 1:** General characteristics of the eight immune-related genes that construct the prognostic model.

Gene symbol	logFC	FDR	Coef	HR	95% CI	*P-*value
SLC10A2	−7.383	9.12E−29	0.633	1.883	1.203−2.948	5.64E−03
UTS2	1.011	2.52E−02	0.333	1.39	1.155−1.674	4.99E−04
FGF2	−1.468	2.62E−13	0.326	1.385	1.155−1.661	4.36E−04
UCN	2.390	4.47E−20	0.283	1.327	1.165−1.512	2.06E−05
IL1RL2	1.160	1.79E−04	0.221	1.248	1.093−1.425	1.05E−03
ESM1	5.974	2.01E−25	0.106	1.112	1.044−1.184	9.47E−04
ADIPOQ	−1.746	9.04E−22	0.087	1.091	1.034−1.151	1.37E−03
VIP	−3.360	1.97E−24	0.044	1.045	1.017−1.075	1.68E−03

### Association Between Prognostic Model and Overall Survival

We obtained data from 413 CRC patients with complete clinical information, including survival status, survival time, age, gender, pathological stage, and TNM stage. As shown in Table 2, detailed clinical characteristics of the patients are listed. The results of the Chi-squared-test suggest that differences in the distribution of the *N* classification in low-risk and high-risk groups were statistically significant. To further confirm the independent predictive power of the prognostic model, univariate and multivariate Cox regression analyses were applied. The univariate analysis showed that age (*P* = 0.005), pathological stage (*P* < 0.001), TNM classification (*P* < 0.001), and the immune-related prognostic model (*P* < 0.001) were significantly correlated with overall survival. The multivariate analysis indicated that immune-related prognostic models (*P* < 0.001), age (*P* < 0.001), and T classification (*P* = 0.008) remained independent prognostic factors related to OS (Table 3).

**TABLE 2:** Clinical characteristics of patients with CRC in different risk groups.^†^

Parameter	Whole cohort (*n* = 413)	Low risk (*n* = 203)	High risk (*n* = 210)	*p*
Age at diagnosis, y				0.489
≤ 60	180 (43.6)	92 (22.3)	88 (21.3)	
> 60	233 (56.4)	111 (26.9)	122 (29.5)	
Gender				0.489
Male	228 (55.2)	116 (28.1)	112 (27.1)	
Female	185 (44.8)	87 (21.1)	98 (23.7)	
Pathological stage				0.071
I	73 (17.7)	43 (10.4)	30 (7.3)	
II	164 (39.7)	86 (20.8)	78 (18.9)	
III	112 (27.1)	47 (11.4)	65 (15.7)	
IV	64 (15.5)	27 (6.5)	37 (9)	
T classification				0.14
T1	12 (2.9)	5 (1.2)	7 (1.7)	
T2	70 (16.9)	43 (10.4)	27 (6.5)	
T3	287 (69.5)	136 (32.9)	151 (36.6)	
T4	44 (10.7)	19 (4.6)	25 (6.1)	
*N* classification				0.014*
N0	244 (59.1)	134 (32.4)	110 (26.6)	
N1	102 (24.7)	44 (10.7)	58 (14)	
N2	67 (16.2)	25 (6.1)	42 (10.2)	
M classification				0.277
M0	349 (84.5)	176 (42.6)	173 (41.9)	
M1	64 (15.5)	27 (6.5)	37 (9)	

*p < 0.05. ^†^Patients with information unavailable on tumor stage (15 patients); M classification (53 patients) were excluded from the comparison.

**TABLE 3:** Univariate and multivariate analyses of overall survival in colorectal carcinoma patients.

Parameter	Univariate analysis			Multivariate analysis		
		
	HR	95% CI	*P*	HR	95% CI	*P*
Age	1.034	1.010–1.059	**0.005**	1.051	1.025–1.077	**8.88E−05**
Gender	1.025	0.632–1.661	0.921	0.835	0.505–1.381	0.483
Pathological stage	2.557	1.931–3.384	**5.39E−11**	1.307	0.546–3.128	0.548
T classification	3.618	2.225–5.883	**2.17E−07**	2.157	1.219–3.817	**0.008**
*N* classification	2.254	1.696–2.996	**2.10E−08**	1.353	0.825–2.221	0.231
M classification	5.272	3.215–8.643	**4.39E−11**	2.077	0.624–6.914	0.233
Prognostic model	1.256	1.189–1.327	**3.21E−16**	1.202	1.131–1.277	**2.56E**−**09**

*Bold values indicate P < 0.05. HR, hazard ratio; CI, confidence interval.*

### Clinical Correlation Analysis

**FIGURE 8:**
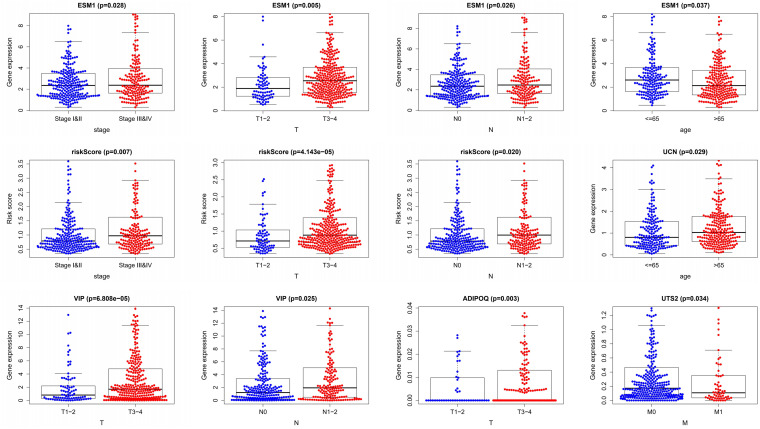
Clinical relevance analysis. Association between the prognostic immune-related signature and clinical factors in CRC.

**FIGURE 9:**
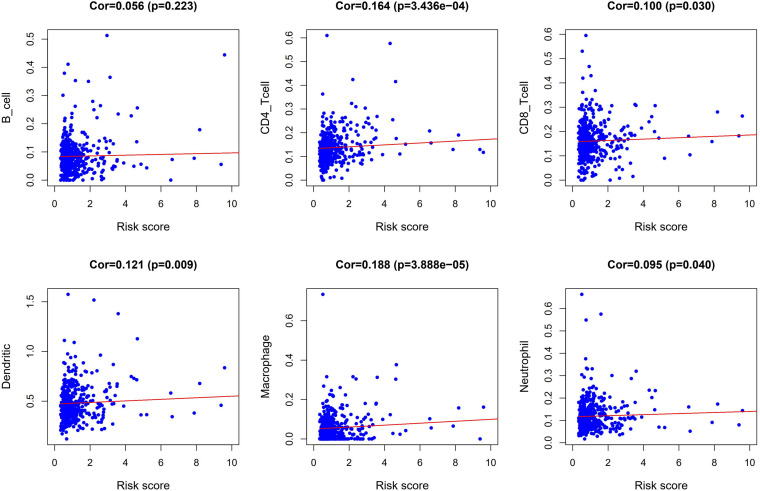
Correlation analysis between risk score and abundances and proportion of six types of immune cell subsets.

We performed a correlation analysis of the immune-related genes involved in the prognostic model and clinical parameters. It was shown that the gene expression of ESM1, VIP, ADIPOQ, UTS2, and UCN were associated with clinical traits. Meanwhile, with the gradual increase of stage and T and *N* classification, the risk score of the prognostic model increased, suggesting the accuracy of the model (Figure 8). The association between the risk score and immune cell infiltration was examined. Results indicated that CD4^+^ T cells (*P* < 0.001), CD8^+^ T cells (*P* = 0.03), dendritic cells (*P* = 0.009), macrophages (*P* < 0.001), and neutrophils (*P* = 0.04) were positively related to the risk score, suggesting that the greater the risk value, the higher the immune infiltration. In addition, B cells had no significant correlation with risk scores (Figure 9).

### ESM1 and SLC10A2 Exert Opposing Roles in Colon Cancer Cell Proliferation

**FIGURE 10:**
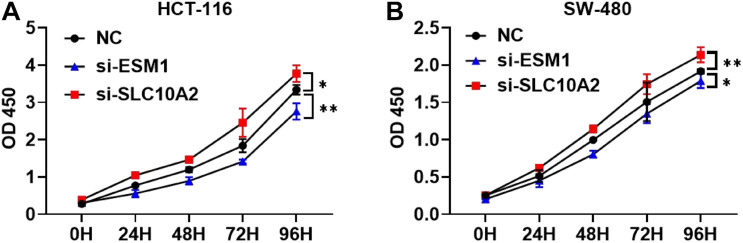
The effects of si-ESM1 and si-SLC10A2 on the proliferation of colon cancer cells. CCK-8 cell proliferation assay results from HCT-116 **(A)** and SW-480 **(B)** cells transfected with si-ESM1, si-SLC10A2, or negative control. *n* = 5 per group, mean values ± SD are presented, *P*-values were calculated using an Unpaired *T*-test, ^∗^*p* < 0.05, ^∗∗^*p* < 0.01.

Furthermore, we attempted to validate the functional effects of ESM1 and SLC10A2 on colon cancer cells *in vitro*. We transfected HCT-116 and SW-480 cell lines with si-ESM1 and si-SLC10A2 or their negative controls (NC). Afterward, cell proliferation was estimated using a CCK-8 assay. Results showed that si-ESM1 inhibits the growth of tumor cells, while si-SLC10A2 promotes the proliferation of tumor cells (Figures 10A,B).

## Discussion

CRC remains one of the most fatal malignant tumors with a poor prognosis globally (Dekker et al., 2019; Gbd. 2017 Colorectal Cancer Collaborators, 2019). Accurate overall survival prediction of CRC is of great significance for selecting therapeutic options and improving the prognosis of patients. However, to date, no robust and effective biomarkers have been identified to accurately predict the survival of CRC patients. The importance of immunity in cancer development and progression is also well recognized (Finotello et al., 2019). Immunotherapy is increasingly important in the treatment of cancer (Helmy et al., 2013; Yang, 2015). Thus, it is necessary to construct an immune-related prediction model for the prognosis of CRC patients.

In this study, 338 immune-related DEGs were screened from 568 samples of CRC and 44 normal tissues. A GO functional analysis revealed that the mentioned genes were mainly related to the regulation of the immune response. KEGG pathways were primarily concentrated on inflammation, immunity, and cancer-related pathways (e.g., cytokine–cytokine receptor interaction, neuroactive ligand–receptor interaction, PI3K-Akt signaling pathway, and NF-kappa B signaling pathway). Forty-five prognostic immune-related DEGs were detected by univariate regression analysis. Furthermore, we created a TF-mediated network to show the regulatory mechanism of the prognostic immune-related DEGs and the key TFs. The results showed that NR3C1 is a key TF node in the network that positively regulated the immune-related genes, such as INHBA, NOX4, SLIT2, LTBP2, PLCG2, COLEC10, and SEMA3G. NR3C1 (nuclear receptor subfamily 3, group C, member 1), also known as a glucocorticoid receptor (GR), was recognized as an epigenetically silenced target gene in CRC (Ahlquist et al., 2008; Wu et al., 2017). Previous studies have suggested that the marker of carcinomas with microsatellite instability were hypermethylated NR3C1 (Ahlquist et al., 2008), its mechanism in the development of CRC may be related to apoptosis (Wei et al., 2016), and further experimental evidence is needed.

We identified eight immune-related genes (SLC10A2, UTS2, FGF2, UCN, IL1RL2, ESM1, ADIPOQ, and VIP) as potential biomarkers of CRC and constructed the prognostic model after a multivariate Cox regression analysis. Next, the risk score was obtained using the model. The accuracy of the model was further demonstrated through the evaluation of the survival analysis, risk analysis, ROC curve, and independent prognostic analysis. Thus, the risk score combined with these eight genes may be a viable clinical predictor of the progression and prognosis in CRC patients.

Compared with normal tissues, UTS2, UCN, IL1RL2, and ESM1 are upregulated in CRC tissues. UTS2 (Urotensin-II) is a somatostatin-like cyclic heptapeptide (Kim et al., 2017) and participates in the occurrence of different cancers, especially colon, breast, and prostate cancers (Giulianelli et al., 2019). It is extensively expressed in vascular endothelial cells and various tissues such as the small intestine, colon, brain, heart, spleen, aorta, and so on (Yumrutas et al., 2015). Several biological processes in tumor biology are regulated by UTS2, including mitogenic, cell proliferation, and angiogenesis (Federico et al., 2017). Urocortin (UCN) is mainly expressed in reproductive organs or urinary systems such as endometrium, testes, epididymis, and urothelial. UCN is a member of the CRH family, and it can inhibit the invasion and metastatic spread of endometrial cancer (Owens et al., 2017). Interleukin-1 receptor-like 2 (IL1RL2), also known as IL-36 receptor, distributed in the intestine, kidney, skin, and brain, is produced by monocytes and T/B lymphocytes. IL1RL2 plays a crucial role in the inflammatory response (Mahil et al., 2017); it is also related to the distant metastasis of breast cancer and the tumor microenvironment (Chen et al., 2019). However, the relationship of these genes with the prognosis of CRC has not been previously studied. Gene functional enrichment analyses show that these genes are significantly enriched in the vascular process in the circulatory system, whereas the majorly enriched pathway is neuroactive ligand–receptor interaction and cytokine–cytokine receptor interaction.

Endothelial cell-specific molecule 1 (ESM1) is secreted by endothelial cells and has been demonstrated to regulate cell adhesion; it can exacerbate inflammatory conditions and enhance tumor proliferation, invasion, and metastasis (Kang et al., 2012). A high expression of ESM1 was reported to be significantly correlated with worse overall survival of CRC and was an independent prognostic parameter of OS (Jiang et al., 2015). This shows that ESM1 has great therapeutic target potential in CRC treatment (Ji et al., 2010).

SLC10A2, FGF2, ADIPOQ, and VIP are under-expressed in CRC. SLC10A2, which encodes an apical sodium-dependent bile acid transporter (ASBT), mainly expressed in the ileum, plays a critical role in cholesterol and bile acid metabolism. It has been previously proven that SLC10A2 gene polymorphisms are related to CRC risk (Wang et al., 2001). In recent years, animal experiments have also proven that a decreased transcriptional expression of SLC10A2 leads to an increase in fecal bile acids and stimulates tumor promotion (Raufman et al., 2015; Aymeric et al., 2018). Fibroblast growth factor 2 (FGF2), widely distributed in the body, is a classic proangiogenic factor (Komi and Redegeld, 2019); it promotes CRC cell proliferation by stimulating angiogenesis and inducing neovascularization (Zhang et al., 2016). The biological function of FGF2 is related to chemotaxis and cell division; it is enriched in the Ras signaling pathway, PI3K-Akt signaling pathway, and Rap1 signaling pathway, affecting the prognosis of CRC. Adiponectin (ADIPOQ) is an adipokine mainly secreted by adipocytes with anti-inflammatory effects (Parida et al., 2019). Studies have found significant associations between a decreased expression level of adiponectin and susceptibility to cancer (Divella et al., 2017). During the development of CRC liver metastasis, decreased adiponectin levels have been observed (Divella et al., 2019). Furthermore, a high level of adiponectin is related to reduced CRC risk (Nimptsch et al., 2017), suggesting that adiponectin may play an important role in the prediction of CRC.

Vasoactive intestinal peptide (VIP) is a neuropeptide that regulates the inflammatory response, and is involved in the pathogenesis of various cancers (Sastry et al., 2017). VIP is expressed in multiple tissues including the nervous system, immune system, digestive system, and can be secreted by lymphocytes, macrophages, and mast cells. Evidence has already shown that VIP can prevent the development of liver cancer through apoptosis of the cAMP/Bcl-xL pathway (Hara et al., 2019), but its prognostic significance in CRC requires further investigation.

Previous studies have shown that neuroactive ligand–receptor interaction pathways interact with the microenvironment cells and tumor cells (Lvu et al., 2020), meanwhile, hypomethylation-high expression genes in CRC were also enriched in the cytokine–cytokine receptor interaction (Liu et al., 2017). The Ras signaling pathway, which is reported to be the most dysregulated pathway in CRC, is associated with cancer cell proliferation, invasion, inflammation, apoptosis, and metastasis (Soleimani et al., 2019). The PI3K/Akt signaling pathway regulates cell survival signals and mediates cell biological progression, and it can affect tumor aggressiveness (Xu et al., 2015). The Rap1 signaling pathway has been shown to be related to endothelial biology and vascular formation (Chrzanowska-Wodnicka, 2013). These results further support the idea that these pathways serve an important role in CRC, which were consistent with the results in our study.

The current study found a positive correlation between the prognostic model and immune cell infiltration. Data indicate that higher infiltration levels of CD4^+^ T cells, CD8^+^ T cells, dendritic cells, macrophages, and neutrophils may be observed in high-risk CRC patients. This demonstrates that the model can be used as an effective tool for an increased immune cell infiltration prediction. Recent research has reported that intratumoral CD4^+^ T cells may be a meaningful predictor of CRC (Kuwahara et al., 2019). CD8^+^ T cells are associated with tumor immune recognition and destruction (Sokol et al., 2015), and the activation of CD8^+^ T cells is increased via dendritic cells (Ziegler et al., 2018), which can be included in immunotherapy against cancer. Macrophages are associated with tumor growth, angiogenesis, and metastasis and are good potential anticancer targets (Wei et al., 2019). Neutrophils contribute to the activation of immune cells, tumor growth, and proliferation (Governa et al., 2017) and can be used as key clinical biomarkers and therapeutic targets.

Our study provides new evidence that immune-related DEGs are associated with the CRC prognosis. The results further confirm and expand the understanding that immune genes and cells regulate CRC progression. An immune-related prognostic model was constructed, providing new insights for the diagnosis and treatment targets of CRC in immunotherapy.

Despite these promising results, the limitations of this study should be taken into consideration. This study utilized retrospective transcriptome analysis data that could not completely reflect the overall immune status. Therefore, clinical data still need to be collected to validate the accuracy of the prognostic model. In addition, further experiments are needed to elucidate the relevant mechanisms of immunogenomics in the occurrence, progression, and metastasis of CRC.

## Conclusion

In summary, we identified numerous immunologically DEGs that are significantly related to the prognosis of CRC. Moreover, we constructed a novel 8-gene immune-related signature that can serve as an independent prognostic indicator for CRC. Meanwhile, the prognostic model can be utilized as a predictor for increased immune cell infiltration. This study contributes to our understanding of immune-related genes in CRC and suggests new potential biomarkers for prognosis and therapy. This will help personalize the use of immunotherapies for CRC patients.

## Data Availability Statement

The datasets presented in this study can be found in online repositories. The names of the repository/repositories and accession number(s) can be found in the article/Supplementary Material.

## Author Contributions

SW and LH finished the major analyses. SW conceived the study and performed the bioinformatics analyses. SW, LH, and ZZ downloaded and organized the clinical and gene expression data. SW, LH, and HM performed the statistical analyses and wrote the manuscript. FL critically revised the article for essential intellectual content. All authors read and approved the final manuscript.

## Conflict of Interest

The authors declare that the research was conducted in the absence of any commercial or financial relationships that could be construed as a potential conflict of interest.

## Footnotes

1 https://portal.gdc.cancer.gov/

2 https://www.immport.org/

3 http://cistrome.org/

4 https://cistrome.shinyapps.io/timer/

## Abbreviations

CRC: colorectal cancer
TCGA: The Cancer Genome Atlas
DEGs: differentially expressed genes
TFs: transcription factor
TIMER: Tumor immune estimation resource
GO: gene ontology database
KEGG: The Kyoto Encyclopedia of Genes and Genomes
ROC: receiver operating characteristic
AUC: area under the ROC curve
OS: overall survival.

## References

[B1] Ahlquist T. Lind G. E. Costa V. L. Meling G. I. Vatn M. Hoff G. S. (2008). Gene methylation profiles of normal mucosa, and benign and malignant colorectal tumors identify early onset markers. *Mol. Cancer.* 7:94. 10.1186/1476-4598-7-94 19117505 PMC2639620

[B2] Aymeric L. Donnadieu F. Mulet C. du Merle L. Nigro G. Saffarian A. (2018). Colorectal cancer specific conditions promote streptococcus gallolyticus gut colonization. *Proc. Natl. Acad. Sci. U.S.A.* 115 E283–E291. 29279402 10.1073/pnas.1715112115 PMC5777054

[B3] Bai J. Zhang X. Xiang Z. X. Zhong P. Y. Xiong B. (2020). Identification of prognostic immune-related signature predicting the overall survival for colorectal cancer. *Eur. Rev. Med. Pharmacol. Sci.* 24 1134–1141. 32096169 10.26355/eurrev_202002_20164

[B4] Bhattacharya S. Dunn P. Thomas C. G. Smith B. Schaefer H. Chen J. (2018). ImmPort, toward repurposing of open access immunological assay data for translational and clinical research. *Sci. Data* 5:180015. 10.1038/sdata.2018.15 PMC5827693 29485622

[B5] Cereda M. Gambardella G. Benedetti L. Iannelli F. Patel D. Basso G. (2016). Patients with genetically heterogeneous synchronous colorectal cancer carry rare damaging germline mutations in immune-related genes. *Nat. Commun.* 7:12072. 10.1038/ncomms12072 PMC4935966 27377421

[B6] Chen Y. C. Gonzalez M. E. Burman B. Zhao X. Anwar T. Tran M. (2019). Mesenchymal stem/stromal cell engulfment reveals metastatic advantage in breast cancer. *Cell Rep.* 27 3916–3926. 10.1016/j.celrep.2019.05.084 31242423 PMC6657699

[B7] Chrzanowska-Wodnicka M. (2013). Distinct functions for Rap1 signaling in vascular morphogenesis and dysfunction. *Exp. Cell Res.* 319 2350–2359. 10.1016/j.yexcr.2013.07.022 23911990 PMC3913003

[B8] Ciardiello D. Vitiello P. P. Cardone C. Martini G. Troiani T. Martinelli E. (2019). Immunotherapy of colorectal cancer: challenges for therapeutic efficacy. *Cancer Treat. Rev.* 76 22–32. 10.1016/j.ctrv.2019.04.003 31079031

[B9] Dekker E. Tanis P. J. Vleugels J. L. A. Kasi P. M. Wallace M. B. (2019). Colorectal cancer. *Lancet* 394 1467–1480. 31631858 10.1016/S0140-6736(19)32319-0

[B10] Divella R. Daniele A. De Luca R. Mazzocca A. Ruggieri E. Savino E. (2019). Synergism of adipocytokine profile and ADIPOQ/TNF-α polymorphisms in NAFLD-associated MetS predict colorectal liver metastases outgrowth. *Cancer Genomics Proteomics* 16 519–530. 10.21873/cgp.20154 31659105 PMC6885361

[B11] Divella R. Daniele A. Mazzocca A. Abbate I. Casamassima P. Caliandro C. (2017). ADIPOQ rs266729 G/C gene polymorphism and plasmatic adipocytokines connect metabolic syndrome to colorectal cancer. *J. Cancer.* 8 1000–1008. 10.7150/jca.17515 28529612 PMC5436252

[B12] Federico A. Zappavigna S. Dallio M. Misso G. Merlino F. Loguercio C. (2017). Urotensin-II receptor: a double identity receptor involved in vasoconstriction and in the development of digestive tract cancers and other tumors. *Curr. Cancer Drug. Targets* 17 109–121. 10.2174/1568009616666160621101248 27338741

[B13] Finotello F. Rieder D. Hackl H. Trajanoski Z. (2019). Next-generation computational tools for interrogating cancer immunity. *Nat. Rev. Genet.* 20 724–746. 10.1038/s41576-019-0166-7 31515541

[B14] Ganesh K. Stadler Z. K. Cercek A. Mendelsohn R. B. Shia J. Segal N. H. (2019). Immunotherapy in colorectal cancer: rationale, challenges and potential. *Nat. Rev. Gastroenterol. Hepatol.* 16 361–375. 10.1038/s41575-019-0126-x 30886395 PMC7295073

[B15] Gbd. 2017 Colorectal Cancer Collaborators. (2019). The global, regional, and national burden of colorectal cancer and its attributable risk factors in 195 countries and territories,1990-2017:a systematic analysis for the Global Burden of Disease Study. *Lancet Gastroenterol. Hepatol.* 4 913–933. 31648977 10.1016/S2468-1253(19)30345-0 PMC7026697

[B16] Giulianelli R. Nardoni S. Bruzzese D. Falavolti C. Mirabile G. Bellangino M. (2019). Urotensin II receptor expression in prostate cancer patients: a new possible marker. *Prostate* 79 288–294. 10.1002/pros.23734 30411388

[B17] Governa V. Trella E. Mele V. Tornillo L. Amicarella F. Cremonesi E. (2017). The interplay between neutrophils and CD8+ T cells improves survival in human colorectal cancer. *Clin. Cancer Res.* 23 3847–3858. 10.1158/1078-0432.ccr-16-2047 28108544

[B18] Hara M. Takeba Y. Iiri T. Ohta Y. Ootaki M. Watanabe M. (2019). Vasoactive intestinal peptide increases apoptosis of hepatocellular carcinoma by inhibiting the cAMP/Bcl-xL pathway. *Cancer Sci.* 110 235–244. 10.1111/cas.13861 30390393 PMC6317926

[B19] Helmy K. Y. Patel S. A. Nahas G. R. Rameshwar P. (2013). Cancer immunotherapy: accomplishments to date and future promise. *Ther. Deliv.* 4 1307–1320. 10.4155/tde.13.88 24116914

[B20] Ji N. Y. Kim Y. H. Jang Y. J. Kang Y. H. Lee C. I. Kim J. W. (2010). Identification of endothelial cell-specific molecule-1 as a potential serum marker for colorectal cancer. *Cancer Sci.* 101 2248–2253. 10.1111/j.1349-7006.2010.01665.x 20735430 PMC11158300

[B21] Jiang H. Fu X. G. Chen Y. T. (2015). Serum level of endothelial cell-specific molecule-1 and prognosis of colorectal cancer. *Genet. Mol. Res.* 14 5519–5526. 10.4238/2015.may.25.3 26125749

[B22] Kang Y. H. Ji N. Y. Han S. R. Lee C. I. Kim J. W. Yeom Y. I. (2012). ESM-1 regulates cell growth and metastatic process through activation of NF-κB in colorectal cancer. *Cell. Signal.* 24 1940–1949. 10.1016/j.cellsig.2012.06.004 22735811

[B23] Keum N. Giovannucci E. (2019). Global burden of colorectal cancer: emerging trends, risk factors and prevention strategies. *Nat. Rev. Gastroenterol. Hepatol.* 16 713–732. 10.1038/s41575-019-0189-8 31455888

[B24] Kim M. Y. Ilyosbek S. Lee B. H. Yi K. Y. Jung Y. S. (2017). A novel urotensin II receptor antagonist, KR-36676, prevents ABCA1 repression via ERK/IL-1β pathway. *Eur. J. Pharmacol.* 803 174–178. 10.1016/j.ejphar.2017.03.056 28363746

[B25] Komi D. E. A. Redegeld F. A. (2019). Role of mast cells in shaping the tumor microenvironment. *Clin. Rev. Allergy Immunol.* 58 313–325. 10.1007/s12016-019-08753-w 31256327 PMC7244463

[B26] Kuwahara T. Hazama S. Suzuki N. Yoshida S. Tomochika S. Nakagami Y. (2019). Intratumoural-infiltrating CD4+and FOXP3+T cells as strong positive predictive markers for the prognosis of resectable colorectal cancer. *Br. J. Cancer.* 121 659–665. 10.1038/s41416-019-0559-6 31488881 PMC6889292

[B27] Leman J. K. Sandford S. K. Rhodes J. L. Kemp R. A. (2018). Multiparametric analysis of colorectal cancer immune responses. *World J Gastroenterol.* 24 2995–3005. 10.3748/wjg.v24.i27.2995 30038466 PMC6054948

[B28] Liu J. Li H. Sun L. Wang Z. Xing C. Yuan Y. (2017). Aberrantly methylated-differentially expressed genes and pathways in colorectal cancer. *Cancer Cell Int.* 17:75. 10.1186/s12935-017-0444-4 PMC5545832 28794688

[B29] Lvu W. Fei X. Chen C. Zhang B. (2020). In silico identification of the prognostic biomarkers and therapeutic targets associated with cancer stem cell characteristics of glioma. *Biosci. Rep.* 40:BSR20201037. 10.1042/BSR20201037 PMC7418212 32725165

[B30] Mahil S. K. Catapano M. Di Meglio P. Dand N. Ahlfors H. Carr I. M. (2017). An analysis of IL-36 signature genes and individuals with IL1RL2 knockout mutations validates IL-36 as a psoriasis therapeutic target. *Sci. Transl. Med.* 9:eaan2514. 10.1126/scitranslmed.aan2514 29021166

[B31] Mei S. Meyer C. A. Zheng R. Qin Q. Wu Q. Jiang P. (2017). Cistrome cancer: a web resource for integrative gene regulation modeling in cancer. *Cancer Res.* 77 e19–e22. 29092931 10.1158/0008-5472.CAN-17-0327 PMC5826647

[B32] Nimptsch K. Song M. Aleksandrova K. Katsoulis M. Freisling H. Jenab M. (2017). Genetic variation in the ADIPOQ gene, adiponectin concentrations and risk of colorectal cancer: a mendelian randomization analysis using data from three large cohort studies. *Eur. J. Epidemiol.* 32 419–430. 10.1007/s10654-017-0262-y 28550647 PMC5535815

[B33] Onyoh E. F. Hsu W. F. Chang L. C. Lee Y. C. Wu M. S. Chiu H. M. (2019). The rise of colorectal cancer in asia: epidemiology, screening, and management. *Curr Gastroenterol Rep.* 21:36. 10.1007/s11894-019-0703-8 31289917

[B34] Or C. H. R. Chang Y. Lin W. C. Lee W. C. Su H. L. Cheung M. W. (2017). Obatoclax, a pan-BCL-2 inhibitor, targets cyclin D1 for degradation to induce antiproliferation in human colorectal carcinoma cells. *Int. J. Mol. Sci.* 18:44. 10.3390/ijms18010044 28035994 PMC5297679

[B35] Owens G. L. Lawrence K. M. Jackson T. R. Crosbie E. J. Sayan B. S. Kitchener H. C. (2017). Urocortin suppresses endometrial cancer cell migration via CRFR2 and its system components are differentially modulated by estrogen. *Cancer Med.* 6 408–415. 10.1002/cam4.967 28109061 PMC5313640

[B36] Parida S. Siddharth S. Sharma D. (2019). Adiponectin, obesity, and cancer: clash of the bigwigs in health and disease. *Int. J. Mol. Sci.* 20:2519. 10.3390/ijms20102519 31121868 PMC6566909

[B37] Patel S. A. Minn A. J. (2018). Combination cancer therapy with immune checkpoint blockade: mechanisms and strategies. *Immunity* 48 417–433. 10.1016/j.immuni.2018.03.007 29562193 PMC6948191

[B38] Raufman J. P. Dawson P. A. Rao A. Drachenberg C. B. Heath J. Shang A. C. (2015). Slc10a2-null mice uncover colon cancer-promoting actions of endogenous fecal bile acids. *Carcinogenesis* 36 1193–1200. 10.1093/carcin/bgv107 26210740 PMC4612337

[B39] Ritchie M. E. Phipson B. Wu D. Hu Y. Law C. W. Shi W. (2015). limma powers differential expression analyses for RNA-sequencing and microarray studies. *Nucleic Acids Res.* 43:e47. 10.1093/nar/gkv007 25605792 PMC4402510

[B40] Sastry K. S. Chouchane A. I. Wang E. Kulik G. Marincola F. M. Chouchane L. (2017). Cytoprotective effect of neuropeptides on cancer stem cells: vasoactive intestinal peptide-induced antiapoptotic signaling. *Cell Death Dis.* 8:e2844. 10.1038/cddis.2017.226 28569785 PMC5520887

[B41] Sokol L. Koelzer V. H. Rau T. T. Karamitopoulou E. Zlobec I. Lugli A. (2015). Loss of tapasin correlates with diminished CD8(+) T-cell immunity and prognosis in colorectal cancer. *J. Transl. Med.* 13:279. 10.1186/s12967-015-0647-1 PMC4551690 26310568

[B42] Soleimani A. Rahmani F. Saeedi N. Ghaffarian R. Khazaei M. Ferns G. A. (2019). The potential role of regulatory microRNAs of RAS/MAPK signaling pathway in the pathogenesis of colorectal cancer. *J. Cell Biochem.* 120 19245–19253. 10.1002/jcb.29268 31512778

[B43] Sun D. Chen J. Liu L. Zhao G. Dong P. Wu B. (2018). Establishment of a 12-gene expression signature to predict colon cancer prognosis. *PeerJ.* 6:e4942. 10.7717/peerj.4942 29915691 PMC6004299

[B44] Thyer L. Ward E. Smith R. J. Branca J. J. V. Morucci G. Gulisano M. (2013). Therapeutic effects of highly purified de-glycosylated gcmaf in the immunotherapy of patients with chronic diseases. *Am. J. Immunol.* 9 78–84. 10.3844/ajisp.2013.78.84

[B45] Wang W. Xue S. Ingles S. A. Chen Q. Diep A. T. Frankl H. D. (2001). An association between genetic polymorphisms in the ileal sodium-dependent bile acid transporter gene and the risk of colorectal adenomas. *Cancer Epidemiol. Biomarkers Prev.* 10 931–936. 11535543

[B46] Wei C. Yang C. Wang S. Shi D. Zhang C. Lin X. (2019). Crosstalk between cancer cells and tumor associated macrophages is required for mesenchymal circulating tumor cell-mediated colorectal cancer metastasis. *Mol Cancer.* 18:64. 10.1186/s12943-019-0976-4 PMC6441214 30927925

[B47] Wei T. T. Lin Y. T. Chen W. S. Luo P. Lin Y. C. Shun C. T. (2016). Dual targeting of 3-hydroxy-3-methylglutaryl coenzyme a reductase and histone deacetylase as a therapy for colorectal cancer. *EBioMedicine.* 10 124–136. 10.1016/j.ebiom.2016.07.019 27448759 PMC5006731

[B48] Wu S. Wu F. Jiang Z. (2017). Identification of hub genes, key miRNAs and potential molecular mechanisms of colorectal cancer. *Oncol. Rep.* 38 2043–2050. 10.3892/or.2017.5930 28902367 PMC5652954

[B49] Xu W. Yang Z. Lu N. (2015). A new role for the PI3K/Akt signaling pathway in the epithelial-mesenchymal transition. *Cell Adh. Migr.* 9 317–324. 10.1080/19336918.2015.1016686 26241004 PMC4594353

[B50] Yang Y. (2015). Cancer immunotherapy: harnessing the immune system to battle cancer. *J. Clin. Invest.* 125 3335–3337. 10.1172/jci83871 26325031 PMC4588312

[B51] Yu G. Wu Y. Wang W. Xu J. Lv X. Cao X. (2019). Low-dose decitabine enhances the effect of PD-1 blockade in colorectal cancer with microsatellite stability by re-modulating the tumor microenvironment. *Cell Mol Immunol.* 16 401–409. 10.1038/s41423-018-0026-y 29622799 PMC6461874

[B52] Yumrutas O. Oztuzcu S. Büyükhatipoglu H. Bozgeyik I. Bozgeyik E. Igci Y. Z. (2015). The role of the UTS2 gene polymorphisms and plasma Urotensin-II levels in breast cancer. *Tumour. Biol.* 36 4427–4432. 10.1007/s13277-015-3082-2 25604143

[B53] Zhang W. Lu X. Cui P. Piao C. Xiao M. Liu X. (2019). Phase I/II clinical trial of a Wilms’ tumor 1-targeted dendritic cell vaccination-based immunotherapy in patients with advanced cancer. *Cancer Immunol. Immunother* 68 121–130. 10.1007/s00262-018-2257-2 30306202 PMC11028035

[B54] Zhang X. Xu J. Jiang T. Liu G. Wang D. Lu Y. (2016). MicroRNA-195 suppresses colorectal cancer cells proliferation via targeting FGF2 and regulating Wnt/β-catenin pathway. *Am J Cancer Res.* 6 2631–2640. 27904776 PMC5126278

[B55] Ziegler P. K. Bollrath J. Pallangyo C. K. Matsutani T. Canli Ö De Oliveira T. (2018). Mitophagy in intestinal epithelial cells triggers adaptive immunity during tumorigenesis. *Cell* 174 88.e16–101.e16. 29909986 10.1016/j.cell.2018.05.028 PMC6354256

[B56] Zuo S. Dai G. Ren X. (2019). Identification of a 6-gene signature predicting prognosis for colorectal cancer. *Cancer Cell Int.* 19:6. 10.1186/s12935-018-0724-7 PMC6321660 30627052

