# Supplementary material for: Prognostic Model of Colorectal Cancer Constructed by Eight Immune-Related Genes
Source: Front Mol Biosci. 2020 Nov 27;7:604252. doi: 10.3389/fmolb.2020.604252 (PMC7729086; doi:10.3389/fmolb.2020.604252)
Supplement: Supplementary file 1 [file Table_1.DOC]

Supplement Table 1 General characteristics of prognostic immune-related genes

| Gene symbol | logFC | FDR | HR | 95%CI | pvalue |
| --- | --- | --- | --- | --- | --- |
| NGF | -1.048 | 4.37E-09 | 2.194 | 1.438-3.348 | 0.000 |
| SLC10A2 | -7.383 | 9.12E-29 | 1.897 | 1.240-2.903 | 0.003 |
| NOX4 | 3.194 | 2.91E-19 | 1.667 | 1.189-2.338 | 0.003 |
| PTH1R | -1.890 | 1.24E-21 | 1.654 | 1.256-2.177 | 0.000 |
| OXTR | 2.976 | 7.77E-24 | 1.392 | 1.141-1.698 | 0.001 |
| TNFRSF13C | -1.518 | 9.84E-10 | 1.378 | 1.092-1.738 | 0.007 |
| PLCG2 | -1.685 | 1.75E-20 | 1.341 | 1.018-1.768 | 0.037 |
| FGF2 | -1.468 | 2.62E-13 | 1.322 | 1.127-1.552 | 0.001 |
| SLIT2 | -1.682 | 2.45E-16 | 1.288 | 1.000-1.658 | 0.050 |
| UTS2 | 1.011 | 0.025 | 1.271 | 1.061-1.523 | 0.009 |
| UCN | 2.390 | 4.47E-20 | 1.241 | 1.088-1.415 | 0.001 |
| CD19 | -1.820 | 1.95E-13 | 1.232 | 1.036-1.465 | 0.018 |
| MC1R | 1.541 | 5.44E-16 | 1.229 | 1.018-1.484 | 0.032 |
| IL1RL2 | 1.160 | 1.79E-04 | 1.220 | 1.068-1.393 | 0.003 |
| KL | -1.292 | 6.18E-18 | 1.180 | 1.007-1.383 | 0.041 |
| SEMA3G | -1.830 | 4.52E-21 | 1.179 | 1.033-1.344 | 0.014 |
| NGFR | -1.742 | 3.55E-19 | 1.172 | 1.058-1.298 | 0.002 |
| GNAI1 | -1.346 | 1.38E-19 | 1.124 | 1.009-1.252 | 0.034 |
| CD79B | -2.021 | 7.27E-19 | 1.097 | 1.002-1.202 | 0.046 |
| GRP | 2.176 | 1.47E-07 | 1.095 | 1.014-1.183 | 0.020 |
| ESM1 | 5.974 | 2.01E-25 | 1.094 | 1.028-1.165 | 0.005 |
| SCG2 | -2.193 | 2.50E-22 | 1.091 | 1.001-1.189 | 0.049 |
| PGF | 1.523 | 1.50E-15 | 1.089 | 1.004-1.181 | 0.040 |
| ADIPOQ | -1.746 | 9.04E-22 | 1.088 | 1.034-1.145 | 0.001 |
| AMH | 4.226 | 4.94E-15 | 1.079 | 1.004-1.159 | 0.039 |
| PLXNA3 | 1.328 | 6.13E-18 | 1.074 | 1.010-1.142 | 0.024 |
| STC1 | 2.160 | 2.11E-17 | 1.061 | 1.011-1.114 | 0.017 |
| LTBP2 | 1.002 | 3.10E-04 | 1.055 | 1.017-1.094 | 0.004 |
| VIP | -3.360 | 1.97E-24 | 1.040 | 1.012-1.068 | 0.004 |
| INHBA | 5.495 | 2.05E-25 | 1.034 | 1.009-1.061 | 0.009 |
| JAG2 | 2.140 | 1.70E-20 | 1.031 | 1.001-1.061 | 0.046 |
| VEGFA | 1.628 | 2.07E-23 | 1.025 | 1.000-1.051 | 0.049 |
| CD70 | 1.832 | 1.83E-04 | 1.016 | 1.001-1.031 | 0.038 |
| FABP4 | -1.660 | 1.55E-20 | 1.012 | 1.006-1.019 | 0.000 |
| SPP1 | 4.019 | 2.91E-14 | 1.001 | 1.000-1.003 | 0.040 |
| CXCL1 | 2.911 | 7.74E-20 | 0.995 | 0.990-0.999 | 0.026 |
| CXCL3 | 2.966 | 1.54E-20 | 0.982 | 0.969-0.996 | 0.010 |
| F2RL1 | -1.131 | 1.48E-20 | 0.981 | 0.963-0.999 | 0.043 |
| BIRC5 | 1.618 | 7.87E-22 | 0.970 | 0.944-0.996 | 0.027 |
| BID | 1.129 | 4.74E-21 | 0.955 | 0.914-0.997 | 0.035 |
| CCL28 | -2.042 | 4.46E-21 | 0.932 | 0.884-0.982 | 0.008 |
| IL13RA2 | 1.503 | 7.32E-06 | 0.654 | 0.432-0.988 | 0.044 |
| CD1B | 1.026 | 0.012 | 0.184 | 0.045-0.756 | 0.019 |
| GLP2R | -3.799 | 2.01E-25 | 0.069 | 0.008-0.605 | 0.016 |
| COLEC10 | 2.777 | 1.88E-05 | 0.036 | 0.001-0.915 | 0.044 |

Supplement Table 2 General characteristics of prognostic TFs

| Gene symbol | logFC | fdr | HR | 95%CI | HR.95L | HR.95H | pvalue |
| --- | --- | --- | --- | --- | --- | --- | --- |
| TCF7L1 | -1.386 | 1.16E-18 | 1.425 | 1.167-1.741 | 1.167 | 1.741 | 5.26E-04 |
| SALL4 | 5.044 | 4.44E-25 | 1.215 | 1.069-1.380 | 1.069 | 1.380 | 0.003 |
| ELF5 | 7.817 | 1.04E-10 | 1.134 | 1.036-1.240 | 1.036 | 1.240 | 0.006 |
| SNAPC4 | 1.036 | 8.08E-19 | 1.106 | 1.010-1.212 | 1.010 | 1.212 | 0.030 |
| WWTR1 | -1.007 | 1.74E-07 | 1.064 | 1.009-1.123 | 1.009 | 1.123 | 0.022 |
| FOSL1 | 2.389 | 1.06E-18 | 1.020 | 1.007-1.034 | 1.007 | 1.034 | 0.003 |
| TEAD4 | 2.441 | 5.51E-25 | 1.013 | 1.002-1.024 | 1.002 | 1.024 | 0.021 |
| FOXM1 | 1.517 | 8.50E-21 | 1.010 | 1.001-1.019 | 1.001 | 1.019 | 0.031 |
| NCAPG | 1.438 | 8.65E-19 | 0.907 | 0.825-0.997 | 0.825 | 0.997 | 0.043 |
